# The ATP-Gated P2X7 Receptor As a Target for the Treatment of Drug-Resistant Epilepsy

**DOI:** 10.3389/fnins.2017.00021

**Published:** 2017-02-02

**Authors:** Edward Beamer, Wolfgang Fischer, Tobias Engel

**Affiliations:** ^1^Department of Physiology and Medical Physics, Royal College of Surgeons in IrelandDublin, Ireland; ^2^Medical Faculty, Rudolf-Boehm-Institute of Pharmacology and Toxicology, University of LeipzigLeipzig, Germany

**Keywords:** epilepsy, seizures, drug-refractory, inflammation, ATP, P2X7 receptor

## Abstract

Despite the progress made in the development of new antiepileptic drugs (AEDs), the biggest challenges that epilepsy presents to drug development have remained unchanged for the last 80 years: finding a treatment with potential for modifying disease progression and reducing the percentage of patients resistant to all pharmacological interventions. The mechanism of action of the majority of AEDs is based on blocking Na^+^ and/or Ca^2+^ channels, promotion of GABA or inhibition of glutamate signaling. In order for further progress to be made, however, a fuller picture of epilepsy will need to be considered, including changes to blood–brain barrier permeability, synaptic plasticity, network reorganization, and gliosis. In particular, brain inflammation has attracted much attention over recent years. Emerging evidence demonstrates a causal role for brain inflammation in lowering seizure thresholds and driving epileptogenesis. Consistent with this, intervening in pro-inflammatory cascades has shown promise in animal models of epilepsy, with clinical trials of anti-inflammatory agents already underway. The ATP-gated purinergic P2X7 receptor (P2X7) has been proposed as a novel drug target for a host of neurological conditions, including epilepsy. Constitutive expression of P2X7 in the CNS is mainly on microglia, but neuronal and astroglial expression has also been suggested. Its function as a gatekeeper of inflammation is most clearly understood, however, it also plays a number of other important roles pertinent to icto- and epileptogenesis: depolarization of the cell membrane, release of macromolecules, induction of apoptosis and synaptic reorganization. Changes in P2X7 expression have been reported following prolonged seizures (status epilepticus) and during chronic epilepsy in both experimental models and patients. While much of the early work focused on the study of P2X7 during status epilepticus, there is now mounting data showing involvement of this receptor during epilepsy. The present short review will discuss the most recent findings concerning P2X7 expression and function during epilepsy and the clinical potential for P2X7 antagonists as novel AEDs.

## Epilepsy

Epilepsy encompasses a complex group of chronic neurological diseases, characterized by the manifestation of recurrent seizures and has an incidence of ~1% with over 60 million people worldwide suffering from the disease (Moshé et al., 2015). Epilepsy affects people of all ages, with incidence highest in the young and elderly (Bialer and White, 2010). Patients with epilepsy have a life expectancy reduced by 2–10% compared with the general population, a death rate 2–3 times higher, and a 4-fold risk of a host of co-morbidities such as depression and anxiety which impact upon quality of life (Moshé et al., 2015). Epilepsy can be acquired as a result of a brain insult, such as head trauma, stroke, or an episode of status epilepticus (SE). Equally, it can result from genetic polymorphisms, copy number variants, or *de novo* mutations, often involving changes in ion channel function (Rees, 2010; Pitkänen and Lukasiuk, 2011). The most common form of acquired epilepsy in adults is temporal lobe epilepsy (TLE), in which seizures arise from brain structures such as the hippocampus and amygdala (Chang and Lowenstein, 2003). Hippocampal sclerosis, characterized by a pattern of selective neuronal loss and reactive gliosis (Chang and Lowenstein, 2003) is the most common pathological finding in the brain of TLE patients.

Epileptogenesis is the process of structural and functional changes which transforms the normal brain to one that can generate the abnormal neuronal activity underlying seizures. Classically, the process of epileptogenesis is understood to occur during the latent period between an initial insult and first spontaneous seizure. More recent concepts of epileptogenesis, however, describe an ongoing process continuing to drive progression of the disease beyond its emergence (Pitkänen and Engel, 2014). Epileptogenesis is characterized by a number of pathological changes, such as delayed, ongoing neurodegeneration, synaptic plasticity, increased blood–brain barrier (BBB) permeability, extracellular matrix reorganization, neurogenesis, and chronic inflammation (Pitkänen and Lukasiuk, 2011). Currently available treatments, as discussed below, act to suppress seizures, but have little impact on the process of epileptogenesis.

## Current treatment

For newly diagnosed patients suffering from epilepsy, antiepileptic drugs (AEDs) are the frontline treatment (Wiebe and Jette, 2012). While the model of epilepsy as an imbalance between excitation and inhibition has served as the backbone for rational drug design, new AEDs have continued to enter the market, improving control of seizures, limiting adverse effects and broadening the available pharmacological armamentarium. This has offered greater scope for physicians to prescribe drugs tailored to the particular needs of a patient, such as avoiding complications with pregnancy (Patel and Pennell, 2016) or the exacerbation of co-morbidities, such as depression (Blond et al., 2016). Across a span of almost 80 years, however, the percentage of patients refractory to all available AEDs has steadfastly refused to move from 30% (Moshé et al., 2015), with TLE particularly resistant to treatment (Pitkänen and Lukasiuk, 2011). While currently there are more than 25 AEDs available on prescription, despite the superficial diversity, all current treatments rely on three main mechanisms of action based on rebalancing excitatory/inhibitory drive: blockade of Na^+^ and/or Ca^2+^ channels, promotion of GABAergic neurotransmission, or accessory antagonism of glutamate receptors (Bialer and White, 2010). Where AEDs do successfully control seizures, they may exacerbate co-morbidities or cause severe adverse effects (Elger, 2016). Further, available AEDs act mainly symptomatically, controlling seizures but having no effect on disease progression. Typically, patients are dependent on drugs for the entire duration of their life. Where they become refractory to treatment, surgical intervention remains, in the majority of cases, the only available avenue (Wiebe and Jette, 2012). There is therefore a pressing need for the development of new treatment strategies with a non-classical mechanism of action, which show efficacy in refractory patients, have a reduced burden of adverse effects, impact upon associated co-morbidities and retard disease progression. In recent years, the paradigm of epilepsy research has broadened, taking into account the importance of brain inflammation as a possible driver of hyperexcitability and neurodegeneration during epileptogenesis (Vezzani et al., 2016).

## Brain inflammation in icto- and epileptogenesis

Brain inflammation, implicated in a host of neurological disorders (Ransohoff, 2016), has been shown to drive increases in BBB permeability (Rochfort and Cummins, 2015), facilitate glutamatergic neurotransmission (Vezzani and Viviani, 2015), initiate pro-apoptotic signaling pathways, promote selective neuronal death and subsequent rewiring of networks, and stimulate astro- and microgliosis (Ransohoff, 2016). Release of pro-inflammatory cytokines, such as interleukin-1ß (IL-1β) and tumor necrosis factor-alpha (TNFα) increases both in experimental models of epilepsy and in patients (Vezzani et al., 2016). Further, numerous experimental and clinical findings demonstrate that brain inflammation plays a key role in the generation of seizures and the pathogenesis of epilepsy (Vezzani et al., 2016). Experiments showing pro-convulsive effects of pro-inflammatory molecules, such as IL-1β (Balosso et al., 2008) or high mobility group box 1 protein (HMGB1; Maroso et al., 2010) provide evidence that inflammation is capable of driving hyperexcitability. Conversely, anticonvulsive effects of drugs which interfere with inflammatory signaling (Vezzani et al., 2000; Balosso et al., 2008; Marchi et al., 2009; Maroso et al., 2010; Bedner et al., 2015) demonstrate the potential for targeting inflammatory signaling pathways in epilepsy.

## ATP and purinergic signaling

ATP, besides its well-established role in cellular energy transfer, also functions as an important intercellular signaling molecule (Burnstock, 2013). ATP signaling is mediated by “purinergic” P2 receptors (P2Rs), classified into two subfamilies: the P2X homo- or heterotrimeric ionotropic receptors and the P2Y seven-transmembrane-spanning metabotropic receptors. P2X receptors are fast acting and have a lower affinity for ATP, whereas P2Y receptors are slower acting and respond to nanomolar ATP concentrations. To date, seven mammalian P2X subunits and eight P2Y receptors have been discovered (Abbracchio et al., 2009). Under physiological conditions, ATP mediates communication between glial cells and neurons, regulating processes such as synaptic transmission, glial Ca^2+^ waves (Burnstock, 2013), and sleep cycles (Chikahisa and Séi, 2011). Following an insult, ATP is released into the extracellular space at higher concentrations. This occurs either through the compromised cell membrane of damaged and apoptotic cells, or through exocytotic or non-exocytotic release (Dale and Frenguelli, 2009; Idzko et al., 2014), where it initiates inflammatory signaling cascades, principally via P2X7 (Volonté et al., 2012; see more details below). Once released, ATP is rapidly metabolized by ectonucleotidases into different breakdown products including ADP, AMP, and adenosine, each provoking different cellular responses through their activation of different purinergic receptors (Abbracchio et al., 2009).

## P2X7 expression and function in the CNS

Constitutive expression of P2X7 in the CNS is largely restricted to microglia, ependymal cells and oligodendrocytes (Skaper, 2011), although, expression in neurons and astrocytes has also been reported (Armstrong et al., 2002; Engel et al., 2012). The principal site for neuronal P2X7 expression seems to be at presynaptic terminals (Miras-Portugal et al., 2003) where it may contribute to the regulation of neurotransmitter release, including GABA and glutamate (Sperlágh et al., 2002). The picture is not entirely clear, however, with contradictory results regarding P2X7 expression and function in both neurons and astrocytes (Sim et al., 2004; Jabs et al., 2007). Recent studies have demonstrated a role for post-transcriptional regulation in mediating cell-type specific changes in P2X7 expression in response to cues in the cellular environment. Jimenez-Mateos et al. (2015) report that microRNA-22 inhibits the translation of *P2rx7* mRNA into protein in response to mild, non-cell death causing seizures.

While thought mainly to be activated under pathological conditions, P2X7 is believed to be important in cytokine release during normal brain functioning, with P2X7-deficient mice showing reduced cytokine production (Solle et al., 2001). Three distinct features of P2X7 equip it for responding to injury or stress in the CNS: relatively low affinity for ATP, slow desensitization dynamics, and the ability to permeablize the cell membrane to molecules up to 900 Daltons in size (Sperlágh and Illes, 2014). The low sensitivity of P2X7, depending on the extracellular Ca^2+^ and Mg^2+^ concentrations, make it less responsive to the micromolar fluctuations in ATP concentration associated with signaling under physiological conditions (Jiang, 2009). This allows the receptor to function mainly in response to the millimolar concentrations of ATP associated with cell death and excitotoxic stress (Fiebich et al., 2014). Following prolonged activation, P2X7 permeabilizes the cell membrane. It is currently unclear whether this mechanism is via the recruitment of other channels, such as pannexin 1 or the dilation of the P2X7 ion channel itself (Idzko et al., 2014). The consequences however, include a reduction in membrane potential, facilitation of glutamate release via exocytotic or non-exocytotic mechanisms, permeability of the membrane to macromolecules, activation of pro-apoptotic signaling cascades (Beamer et al., 2016), reduction of the action potential threshold via molecular changes at the axon initial segment (Del Puerto et al., 2015), and, as will be described in detail below, initiation of inflammatory cascades (Beamer et al., 2016). Increases in membrane permeability also facilitate the release of ATP itself (Suadicani et al., 2006), though the extent of the contribution of the P2X7 to the release of its own ligand is yet to be determined. All of these processes have the potential to contribute to an epileptic phenotype. Interestingly, P2X7 expression seems to be closely associated with ectonucleotidase tissue-non-specific alkaline phosphatase (TNAP) activity, with mice deficient in TNAP showing decreased P2X7 expression. Furthermore, deficiency in TNAP leads to the development of recurrent seizures which are partially mediated by P2X7 (Sebastián-Serrano et al., 2016).

## P2X7 in brain inflammation

P2X7 regulates a variety of signaling pathways contributing to inflammation, with downstream effectors likely to be cell-type dependent. Much of the work performed delineating P2X7-signaling has been carried out on peripheral immune cells and in transfected human embryonic kidney 293 (HEK-293) cell lines, but evidence for P2X7 involvement in inflammatory pathways in the CNS is increasing (Beamer et al., 2016; Burnstock, 2016). The principal, and best elucidated, mechanism by which P2X7 contributes to neuroinflammation is via the activation of the NLRP3 inflammasome; a protein complex consisting of caspase-1, apoptosis-associated speck-like protein containing a CARD (ASC), and nod-like receptor protein 3 (NLRP3; Volonté et al., 2012). Mechanisms for P2X7-mediated inflammasome activation include facilitating increases in cell membrane permeability which may trigger inflammasome activation via the resulting decrease in intracellular K^+^ concentration (Muñoz-Planillo et al., 2013). The inflammasome initiates the cleavage of precursor interleukin molecules into the mature leaderless cytokines, IL-1β, and IL-18, prior to their release into the extracellular space (Beamer et al., 2016). Evidence has also accumulated for ATP-driven, P2X7-dependent release of reactive oxygen species (ROS) from mitochondria, particularly in microglia (Apolloni et al., 2013). Other pathways in which P2X7 activation contributes to the neuroinflammatory response may include inflammasome-independent release of prostaglandin E2 (PGE2) or the activation of membrane metalloproteinases, such as activity of disintegrin-like metalloproteinase 10 (ADAM10) and ADAM17, leading to the removal of chemokine (C-X-C motif) ligand 16 (CXCL16), cluster of differentiation 44 (CD44), soluble amyloid precursor protein (APP), and the IL-6 receptor from the cell membrane, thereby decreasing the sensitivity of the cell to anti-inflammatory mediators (Beamer et al., 2016).

## P2X7 as a drug target in epilepsy

While interest in the importance of purinergic signaling during seizures and epilepsy is increasing, the majority of studies, to date, have focused on the contribution of the ionotropic P2X subfamily, in particular, P2X7 (Engel et al., 2016; Rassendren and Audinat, 2016). Much of the early work trying to establish the role of P2X7 in ictogenesis and epileptogenesis was limited to the description of expressional changes of the receptor during and after seizures using techniques such as Western blotting and immunohistochemistry (Vianna et al., 2002; Rappold et al., 2006; Doná et al., 2009). Since 2010, however, there has been an explosion of data re-examining expressional changes of the receptor after SE and during epilepsy and to determine the functional contribution of P2X7 to seizures (Engel et al., 2016). To establish whether P2X7 blockade alters seizure severity and seizure-induced pathology, the animal models of choice have been mouse models of chemically-induced SE [e.g., by kainic acid (KA) or pilocarpine; Engel et al., 2016]. More recent studies have now also evaluated the effects of P2X7 antagonists in traditional seizure models such as the pentylenetetrazol (PTZ)- and maximal electroshock (MES) seizure threshold test as well as the PTZ-kindling model in rats (Fischer et al., 2016). There is now ample data available demonstrating the impact of P2X7 antagonism on seizure pathology during SE. This has already been extensively reviewed and will not be discussed here (Engel et al., 2016). Over recent years, the focus of research has shifted toward the study of P2X7 signaling during the process of epileptogenesis and epilepsy. This has mainly been due to technical advances in the field, such as the development of more specific, BBB-penetrating, and more brain stable P2X7 antagonists (Rech et al., 2016).

## P2X7 expression in epilepsy

Data showing increased expression of P2X7 during epilepsy has been obtained from both experimental models of epilepsy and patients. Furthermore, the recent use of transgenic *P2rx7*-GFP reporter mice coupled with GFP-guided patch-clamp (Engel et al., 2012; Jimenez-Pacheco et al., 2016) has made it possible to establish the cell-specific expression pattern of P2X7 without relying solely on the use of antibodies (Sim et al., 2004). In one of the first studies using a rat model of pilocarpine-induced epilepsy, Vianna et al. (2002) provided evidence for an increase in hippocampal P2X7 expression, with strong P2X7 immunoreactivity in mossy fibers. These results were later added to by the same group in a second study showing increased hippocampal P2X7 immunoreactivity in microglia and glutamatergic nerve terminals (Doná et al., 2009). Elevated P2X7 protein levels have also been shown in the hippocampus and cortex in the intra-amygdala (i.a.) KA mouse model of epilepsy (Jimenez-Pacheco et al., 2013, 2016), in surgically resected hippocampus and neocortex from drug-refractory TLE patients (Jimenez-Pacheco et al., 2016) and in neocortical nerve terminals of TLE and non-TLE epilepsy patients (Barros-Barbosa et al., 2016). By using *P2rx7*-GFP reporter mice, our group has also now determined cell-specific patterns of *P2rx7* transcription during epilepsy. In the i.a. KA model, cortical and hippocampal *P2rx7* induction was mainly restricted to neurons and microglia. In the cortex, GFP induction was predominantly present in cortical layers V and VI (Jimenez-Pacheco et al., 2013), while in the hippocampus, increased GFP was most prominent in the hippocampal subfield CA1 followed by the dentate gyrus and subfield CA3 (Jimenez-Pacheco et al., 2016). These results have been confirmed by GFP-guided patch-clamp showing increased P2X7 currents in GFP-positive cells when compared to GFP-negative cells from the same animal (Jimenez-Pacheco et al., 2016). This step rules out possible artifacts due to the genetic approach used. In the same study, P2X7 expression was increased in synaptosomes from epileptic mice and showed altered calcium responses when challenged with P2X7 agonists (Jimenez-Pacheco et al., 2016). As observed previously in the i.a. KA model (Engel et al., 2012), GFP reporter activity in epileptic mice was absent in astrocytes, suggesting P2X7 is not increased in these glial cells during epilepsy (Jimenez-Pacheco et al., 2013, 2016).

## P2X7 during epileptogenesis

**Figure 1:**
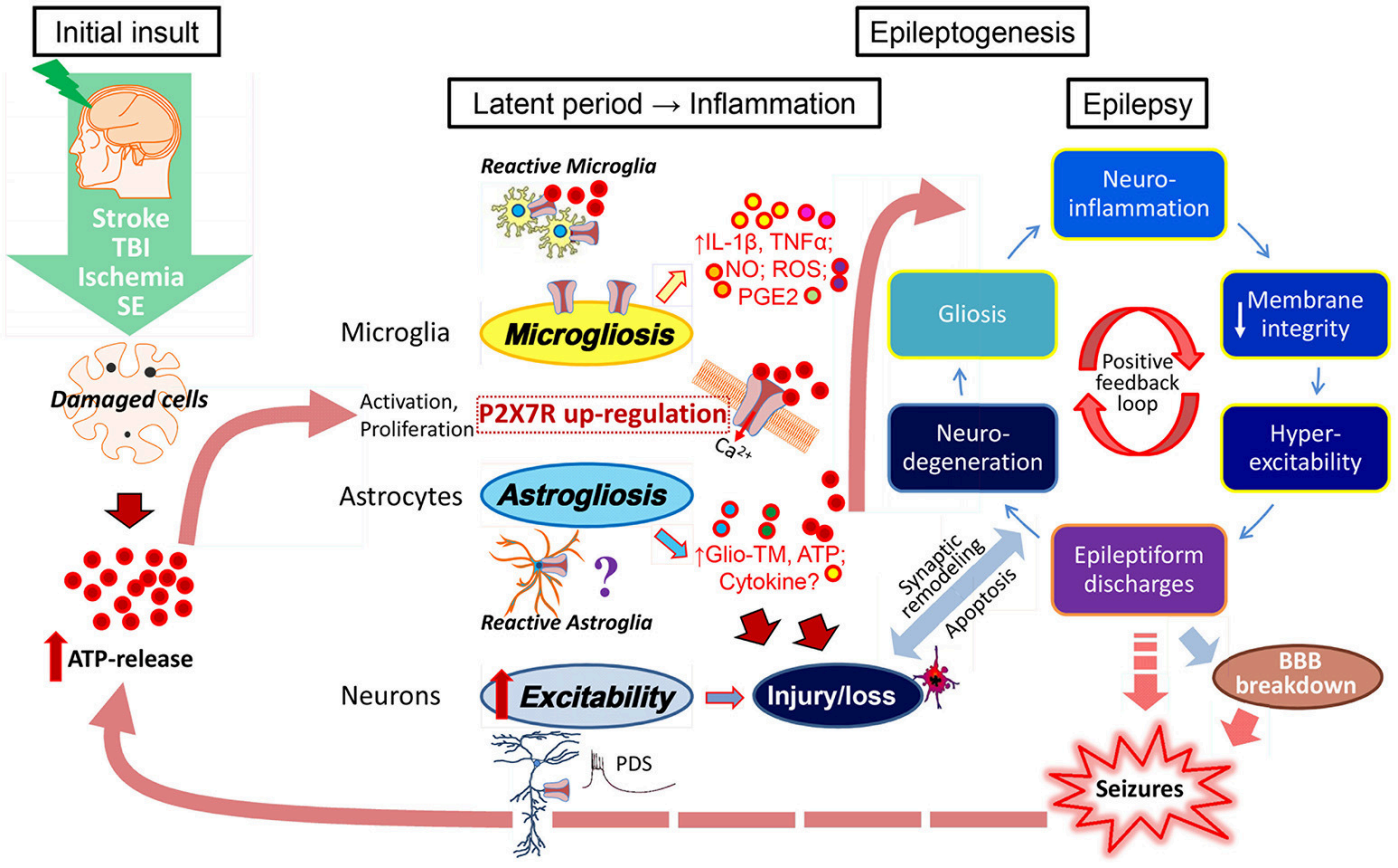
**ATP-driven P2X7 activation as possible contributor to epileptogenesis and epilepsy**. Brain inflammation has been suggested as a crucial etiopathogenic mechanism of epilepsy contributing to seizure generation and the development of epilepsy (Vezzani et al., 2016). Brain injury (stroke, trauma, ischemia)-induced cell damage and/or cell death associated with an up-regulation of P2X7 on microglia and possibly neurons, leads to a massive release of ATP which then acts as a danger signal resulting in the activation of astrocytes and microglia. The P2X7 in particular has been described as a major regulator of the synthesis and secretion of cytokines (IL-1ß, TNFα) and other inflammatory mediators (NO, ROS, PGE2) via microglial cells. However, the P2X7 may also trigger the release of gliotransmitters (Glio-TM) and ATP from astrocytes and neuronal terminals. Continuing brain inflammation is characterized by astro- and microgliosis with enhanced release of ATP and pro-inflammatory mediators/molecules causing changes in neuronal membrane integrity, modifications of ion channels and consequently neuronal hyperexcitability. This will lead to increased brain susceptibility to seizures initiated by paroxysmal depolarization shifts (PDS), rhythmic burst firing, and epileptiform discharges finally producing focal or generalized seizures. Recurrent seizures *per se* promote the release of ATP and pro-inflammatory cytokines and activate immune response that sustains seizure recurrence leading to a vicious cycle of increased inflammation and hyperexcitability. Other pathological mechanism may be involved in epileptogenesis and seizure initiation, such as injury and/or loss of neurons, synaptic remodeling (mossy fiber sprouting), BBB breakdown, and lymphocyte accumulation.

What is the mechanistic link between P2X7 activation and epileptogenesis? ATP, released in high concentrations from damaged cells after an initial brain insult such as stroke, brain trauma, ischemia, infection, or seizures themselves, may act as an acute “danger signal,” activating microglia (and possibly astrocytes and/or neurons) leading to the cleavage and release of mature, leaderless pro-inflammatory cytokines, in particular IL-1β, and other inflammatory molecules (Dale and Frenguelli, 2009; Rodrigues et al., 2015). Continuing brain inflammation may then promote astro- and microgliosis enhancing release of ATP and other gliotransmitters associated with a further increase (possibly via P2X7 upregulation) of the production of various pro-inflammatory cytokines (including IL-1ß, IL-6, and TNFα), danger molecules (e.g., HMGB1, S100ß protein), and other inflammatory mediators (e.g., nitric oxide, ROS, and PGE2). Alterations in membrane permeability, modifications of ion channel function, changes in glutamate receptor subunit expression or reduction of GABA-mediated inhibition, may, in turn, promote neuronal hyperexcitability and, as a consequence, rhythmic burst firing and epileptiform discharges, finally culminating in focal or generalized seizures (Di Maio, 2014). Injury and/or loss of neurons and synaptic remodeling (mossy fiber sprouting), breakdown of the BBB, lymphocyte accumulation and angiogenesis, all processes associated with an increase in P2X7 activity (Sperlágh and Illes, 2014), may also contribute to epileptogenesis and seizure initiation (Friedman and Dingledine, 2011). Chronic inflammation and recurrent seizures *per se* may promote the release of ATP and pro-inflammatory cytokines and activate immune responses which sustain seizure recurrence. This leads to a vicious cycle of P2X7 activation, inflammation, and lowering of the seizure threshold (Librizzi et al., 2012; Figure 1).

The first evidence for the antiepileptogenic potential of P2X7 antagonism was produced using the PTZ-kindling model in rats, a well-established model of epileptogenesis (Soni et al., 2015). In this model, repeated injections of PTZ, with an initial sub-convulsive dose, induce a progressive increase in seizure activity, culminating in the development of generalized tonic-clonic seizures (Fischer and Kittner, 1998; Dhir, 2012). Treatment with the P2X7 antagonist BBG significantly decreased the mean kindling score and restored behavioral deficits, including cognition and motor coordination (Soni et al., 2015). This effect was potentiated by the co-administration of ceftriaxone via up-regulation of the glutamate transporter, GLT-1. Drug washout experiments, however, were not performed. In a more recent study, also using the PTZ-kindling model in rats, the two potent and brain-permeable P2X7 antagonists, JNJ-47965567 and AFC-5128 (15 mg/kg s.c. 30 min, or 30 mg/kg i.p. 45 min before PTZ, respectively), significantly delayed kindling development. This effect was long-lasting, however, the compounds were unable to prevent or reverse the process of epileptogenesis (Fischer et al., 2016). In another study using the i.a. KA model, we found that hippocampal P2X7 up-regulation, achieved through the inhibition of a P2X7-suppressing microRNA (microRNA-22), resulted in a more severe epileptic phenotype (Jimenez-Mateos et al., 2015). Increased P2X7 expression was accompanied by increased cytokine levels (IL-1β and TNFα), astrogliosis and cognitive impairment. Interestingly, microgliosis was reduced in these mice (Jimenez-Mateos et al., 2015). In contrast to these results, the blockade of P2X7 by AZ10606120 (3 μg i.c.v. 30 min post-pilocarpine) or BBG (50 mg/kg i.p. 30 min post-pilocarpine; repeated once per day for 4 days) increased the number of seizures and their severity in rats observed for the 28 following days post-pilocarpine-induced SE (Rozmer et al., 2016), but P2X7 brain occupancy studies were not performed. This is somewhat surprising as AZ10606120 administered shortly after the induction of seizures revealed marked neuroprotective effects in hippocampal neurons (Rozmer et al., 2016), and previous studies by our group using the i.a. KA model have shown that protecting the brain from seizure-induced cell death resulted in a less severe epileptic phenotype (Engel et al., 2010). We do not know what the reasons for the observed differences are. As seen before for SE studies (Kim and Kang, 2011), however, P2X7 antagonism seems to provoke the opposite effect when applied to the KA or pilocarpine model, likely due to differences in the mechanism of induction. Moreover, differences in cell death, limited in the pilocarpine-induced SE mouse model (Engel et al., 2016), may result in differences in the availability of extracellular ATP. Differences in drugs, doses of drugs and route of delivery may also add to variation between studies.

## P2X7 during epilepsy

While increased P2X7 protein levels during epilepsy have been widely reported (Engel et al., 2016), until recently, no evidence existed for an impact of P2X7 antagonism on the epileptic phenotype at all and, therefore, whether P2X7 blockade represents a possible new treatment strategy for epilepsy. Two new studies however, both published in 2016 (Amhaoul et al., 2016; Jimenez-Pacheco et al., 2016), have now attempted to shed some light on this unresolved issue (Table 1). Both studies used KA as a trigger for epilepsy; while one study used the multiple low-dose KA model in rats (Amhaoul et al., 2016), the other, using the i.a. KA model in mice (Jimenez-Pacheco et al., 2016). To investigate the impact of P2X7 antagonism in chronically epileptic rats, the specific P2X7 antagonist JNJ-42253432 (0.6 g/kg/2 ml) was administered via subcutaneous (s.c.) injection for 1 week via mini-pumps in rats, 3 months following KA-induced SE. Epileptic rats treated with JNJ-42253432 experienced the same number of seizure episodes during treatment, however, the severity of these episodes was reduced. The authors concluded that rather than suppressing seizures, P2X7 antagonism leads to a shift in seizure severity, resulting in a milder epileptic phenotype. P2X7 blockade did not alter microgliosis in their study and no drug washout was performed to assess whether changes in the seizure phenotype persists following drug withdrawal (Amhaoul et al., 2016). In the second study, to test whether P2X7 blockade could ameliorate the epileptic phenotype in mice, epileptic mice were treated with another specific P2X7 antagonist, JNJ-47965567 (30 mg/kg i.p.), twice daily for 5 days, followed by a 5-day washout period. In this study, JNJ-47965567 treatment reduced the total amount of epileptic seizures by over 50% during the drug phase. Seizure severity, however, was unchanged. Remarkably, rather than returning to baseline, seizure rates in treated mice continued to decrease during the washout period implying that P2X7 antagonism could modify disease progression (Jimenez-Pacheco et al., 2016). This is even more outstanding as seizure rates in epileptic animals treated with conventional AEDs or anti-inflammatory drugs, if effective, returned to baseline immediately after drug cessation (Grabenstatter et al., 2007; Maroso et al., 2010; Klein et al., 2015). In addition, P2X7 antagonist-treated mice demonstrated a strong reduction in astrogliosis and microgliosis, even when analyzed after washout (Jimenez-Pacheco et al., 2016). We do not know how P2X7 blockade reduces epileptic seizures during and beyond treatment. While effects during treatment may be related to the reduction of neuronal excitability by changes in intracellular ion concentrations or changes in neurotransmitter release (Barros-Barbosa et al., 2016), the most likely explanation for the observed disease-modifying effect is a reduction in astrogliosis and microgliosis. P2X7 has been shown to activate astrocytes. Astrogliosis itself can contribute to a lowering of the seizure threshold via dysregulation of extracellular ionic balance, impaired neurotransmitter reuptake, and release of pro-inflammatory cytokines and purines, including ATP and adenosine (Bedner et al., 2015; Robel et al., 2015). P2X7 has also been shown to drive microglial activation directly, thereby increasing the release of cytokines such as IL-1β (Monif et al., 2009). Chronic brain inflammation in turn leads to increased extracellular concentrations of ATP and P2X7 activity. P2X7 antagonists may therefore act as a break interrupting the vicious cycle of increased neuroinflammation and hyperexcitability (Figure 1).

**Table 1:** **Summary of the main findings related to P2X7 expression and function during epileptogenesis and epilepsy in experimental models of epilepsy and patients**.

**Process**	**Epilepsy model/Patients**	**Techniques and P2X7 antagonists**	**Main results**	**References**
Epileptogenesis	PTZ kindling (30mg/kg i.p every second day for 27 days) in rats	Seizure behavior; Rotarod; Morris Water Maze; Object recognition; BBG (15 and 30mg/kg i.p.) 30 min before PTZ injection	P2X7 blocking reduced seizure score and improved motor performance and cognitive deficits	Soni et al., 2015
Epileptogenesis	i.a. KA-induced epilepsy in mice	GFP-*P2rx7* reporter mouse; patch-clamp; WB; EEG; P2X7-regulating microRNA-22 blockade	Increased P2X7 levels and function leads to increased seizure frequency and increased inflammation (astrocytosis)	Jimenez-Mateos et al., 2015
Epileptogenesis	Pilo i.p.- and KA i.p.-induced epilepsy in mice and rats	Seizure behavior, IH; one single AZ10606120 (3 μg/2μl i.c.v.) post-SE or BBG (50mg/kg i.p.) 1 injection per day for 4 days post-SE	P2X7 blockade prevented neuronal degeneration after SE, but increased the number and severity of seizures during epilepsy	Rozmer et al., 2016
Epileptogenesis	PTZ kindling (35mg/kg i.p.) in rats for 25 days; MES-T and PTZ-T test in mice	Ca^2+^ fluorometry; RT-PCR; WB; IH; JNJ-47965567 (15), AFC-5128 (30), BBG (50), tanshinone (30mg/kg i.p.) before PTZ	P2X7 blocking reduced kindling development and glial activation; none of the compounds revealed anticonvulsant effects in the acute seizure tests in mice	Fischer et al., 2016
Epilepsy	Pilo i.p.-induced epilepsy in rats	Ca^2+^ fluorometry; WB; IH	Abnormal biphasic response to ATP (short increase followed by abrupt decrease); increased expression of P2X7 and mossy fiber sprouting (HIP) during epilepsy	Vianna et al., 2002
Epilepsy	Pilo i.p.-induced epilepsy in rats	WB; IH immunohistochemistry	Diffuse P2X7 expression almost exclusively in nerve terminals during epilepsy	Doná et al., 2009
Epilepsy	i.a. KA-induced epilepsy in mice; TLE epilepsy patients	WB; GFP-*P2rx7* reporter mouse	Increased P2X7 expression in neurons and microglia in cortex in mice; increased P2X7 expression in cortex in TLE patients	Jimenez-Pacheco et al., 2013
Epilepsy	MTLE and non-MTLE patients	WB; IH; neurotransmitter up-take experiments in isolated nerve terminals	Increased P2X7 levels in neocortical nerve terminals in epilepsy patients; P2X7 activation down-modulates GABA uptake by neocortical nerve terminals of epileptic patients	Barros-Barbosa et al., 2016
Epilepsy	Multiple, low-dose KA (total KA = 22.2 ± 2.02 mg/kg i.p.) induced epilepsy in rats	Seizure behavior; EEG; JNJ-47965567 during 1 week via osmotic mini-pump (0.6 g/kg/2 ml)	P2X7 blocking led to decreased seizure severity, however, no change in total numbers of seizures; no change in inflammation after P2X7 antagonist treatment	Amhaoul et al., 2016
Epilepsy	i.a. KA-induced epilepsy in mice; TLE epilepsy patients	GFP-*P2rx7* reporter mouse; patch-clamp; WB; RT-PCR; EEG; seizure behavior; JNJ-47965567 during epilepsy (30mg/kg twice daily for 5 days)	Increased expression of P2X7 in microglia and neurons (HIP); increased P2X7 function in synaptosomes; increased P2X7 levels in TLE patients (HIP); reduced seizure frequency during P2X7 inhibitor treatment and during washout period; P2X7 blocking decreased inflammation (astrogliosis and microgliosis)	Jimenez-Pacheco et al., 2016

*BBG, brilliant blue G; EC, entorhinal cortex; EEG, electroencephalogram; HIP, hippocampus; IH, immunohistochemistry and/or immunofluorescence; KA, kainic acid; MES-T, maximal electroshock seizure threshold test; MTLE, mesial temporal-lobe epilepsy; Pilo, pilocarpine; PTZ-T, pentylenetetrazol seizure threshold test; RT-PCR, quantitative real-time polymerase chain reaction; SE, status epilepticus; TLE, temporal-lobe epilepsy: TNFα, tumor necrosis factor-alpha; WB, Western blot*.

## Conclusions and future directions

Mounting data obtained from both experimental animal models and patients has now convincingly demonstrated a causal role for P2X7 signaling during seizures and epilepsy. Whether P2X7 antagonism has an anticonvulsive or neuroprotective effect, however, is still not completely understood and seems to depend, at least in part, on the animal model used. While mixed results have been obtained regarding the effect of P2X7 antagonists when administered prior to or soon after the induction of SE, the effect of administration following the emergence of chronic epilepsy seems to consistently ameliorate the epileptic phenotype. How to bring P2X7 antagonists further toward a possible clinical application? The development of BBB permeable, brain stable and highly specific P2X7 antagonists has been an important step forward, however, several urgent questions remain to be resolved. (i) Adverse effects of P2X7 antagonism seem to be pertinent to the pilocarpine model, however, we must dissect why P2X7 antagonism results in a different phenotype according to the animal model used. (ii) We still do not know when ATP is released and what local concentrations are reached. Attempts have been made (Doná et al., 2016), however, the techniques used may lack sufficient temporal resolution to detect seizure-induced ATP release in the brain. (iii) What are the cell-specific contributions of P2X7 to disease progression? Various publications have suggested an upregulation of the receptor in neurons, however, the consequences of this up-regulation are unknown. (iv) Does P2X7 blockade lead to neuroprotection or is the reduction in cell death observed during SE a mere consequence of their anticonvulsive properties? (v) How can patients who could potentially benefit from P2X7 blockade be identified? Are there any biomarkers which would predict a pathological P2X7 activation in the brain? (vi) What is the optimal treatment regimen, dose and time window for application? Previous studies have suggested that a positive outcome may depend on the time-point of intervention (Roth et al., 2014; Kaiser et al., 2016). (vii) Finally, do P2X7 antagonists work where other AEDs fail, or show synergistic effects with these compounds? Could they have utility as adjunctive treatment in conjunction with current AEDs as demonstrated in the i.a. KA model and MES seizure threshold test (Engel et al., 2012; Fischer et al., 2016)?

In conclusion, while a possible benefit for P2X7 antagonism during acute seizures and epileptogenesis remains controversial, recent research has shown the potential of P2X7 antagonists for the treatment of epilepsy, thereby providing a much needed new target with a new mechanism of action distinct from currently used AEDs. The availability of new tools and drugs will hopefully shed light on outstanding unanswered questions and accelerate progress toward possible future clinical use.

## Author contributions

EB, wrote the manuscript; WF, designed the figure and edited the manuscript; TE, wrote and edited the manuscript.

### Conflict of interest statement

The authors declare that the research was conducted in the absence of any commercial or financial relationships that could be construed as a potential conflict of interest.

## Abbreviations

BBB: blood–brain barrier
IL-1ß: interleukin-1ß
NO: nitric oxide
PGE2: prostaglandin E2
ROS: reactive oxygen species
SE: status epilepticus
TBI: traumatic brain injury
TNFα: tumor necrosis factor-alpha.
